# Extirpation of the Larynx

**Published:** 1886-05

**Authors:** 


### Extirpation of the Larynx.

Dr. J. Baratoux gives, in the *Progrès Médical* for March 27, 1886, a tabular statement of the cases of this operation performed up to the date of his writing, March 1, 1886. The larynx was first extirpated successfully by Watson, of Edinburgh, in 1866. He was followed by Billroth in 1873, and one hundred operations have since been performed. Three cases have been operated on in Ameria, one each by Lange and Gerster, of New York, and by Roswell Park, of Buffalo.

Of the 102 operations, 73 were for cancer or epithelioma, 10 for sarcoma, 10 for necrosis, stenosis or polypus, and 9 for unascertained causes. In 97 cases the result is known, 83 being cases of total extirpation and 14 of partial.

TOTAL EXTIRPATIONS.

| Lesion. | No. of Cases. | Recovered. | Died. | Unknown. |
|---|---|---|---|---|
| Epithelioma or Cancer, | 69 | 21 | 47 | 1 |
| Sarcoma, . . . . . | 9 | 2 | 7 | |
| Stricture, Necrosis, . | 4 | 1 | 3 | |
| Unknown, . . . . | 1 | | 1 | |
| Total, . . . . . | 83 | 24 | 58 | 1 |

PARTIAL EXTIRPATIONS.

| Lesion. | Cases. | Recovered. | Died. |
|---|---|---|---|
| Epithelioma, . . . . . . . . . | 4 | 2 | 2 |
| Sarcoma, . . . . . . . . . . | 1 | 1 | |
| Stricture, Necrosis, . . . . . . | 6 | 2 | 4 |
| Unknown, . . . . . . . . . . | 3 | 3 | |
| Total, . . . . . . . . . . | 14 | 8 | 6 |

These tables show that the mortality following this operation is just two-thirds, there having been 32 recoveries and 64 deaths.

---

Missouri is to have a crematorium, located, probably, in St. Louis.

